# Analysis of the *SLC4A1* gene in three Mexican patients with hereditary spherocytosis: Report of a novel mutation

**DOI:** 10.1590/S1415-47572009005000109

**Published:** 2010-03-01

**Authors:** Josefina Y. Sánchez-López, Ana L. Camacho-Torres, Bertha Ibarra, Jesús A. Tintos, Francisco J. Perea

**Affiliations:** 1 División de Genética, Centro de Investigación Biomédica de Occidente, Instituto Mexicano del Seguro Social, Guadalajara, JaliscoMexico; 2 Centro Universitario de Ciencias de la Salud, Universidad de Guadalajara, Guadalajara, JaliscoMexico

**Keywords:** hereditary spherocytosis, hemolytic anemia, SLC4A1 gene, AE1 protein, band 3

## Abstract

We analyzed the *SLC4A1* gene in three Mexican patients with Hereditary Spherocytosis (HS). The promoter and all 20 exons were investigated through heteroduplex analysis and DNA sequencing. No DNA changes were detected in one of the three patients. Two well-known polymorphisms, Memphis I and the Diego-a blood group, were detected in another one. In the third, the HS phenotype could be explained by the novel 1885_1888dupCCGG mutation found in heterozygosis. This frameshift mutation is predicted to result in a truncated and unstable protein lacking normal functions.

Hereditary spherocytosis (HS) is a variably severe form of hemolytic anemia, caused by defects in the components of red cell membranes. It is characterized by the presence of spherical, dense and osmotically fragile red cells, which are selectively trapped in the spleen (Gallagher, 2005).

Spectrins, ankyrins, 4.2 protein and anion exchanger 1 (AE1) are the main defective proteins in subjects with HS (Delaunay, 2002). In a few studies of HS in Mexico, the main defective proteins observed were spectrins and AE1 (Sánchez-López *et al.*, 2003).

The *SLC4A1* gene (solute carrier family 4, anion exchanger, member 1) belongs to the anion-exchanger family, and encodes two AE1 isoforms, namely erythroid (eAE1) and renal (rAE1). The eAE1 isoform is a glycoprotein of 911 amino acids with three domains: (1) the N-terminal cytoplasmic domain (residues 1-403), containing the binding sites for hemoglobin and some cytosolic enzymes, which acts as a membrane anchorage site for the red cell skeleton through its interactions with ankyrin, and 4.1 and 4.2 proteins; (2) the transmembrane domain (residues 404-882), which has 14 segments spanning the lipid bilayer and is responsible for Cl^–^/HCO_3_^–^ exchange; and (3) a short cytoplasmic C-terminal domain (residues 883-911), containing binding sites for carbonic anhydrase II. The rAE1 isoform lacks the first 65 amino acids of the N-terminal domain, since it is transcribed from a second promoter located in intron 3 of the *SLC4A1* gene (Alper, 2006; Delaunay, 2002).

Several mutations of *SLC4A1* have been described that result in distal renal tubular acidosis (Bruce *et al.*, 1997; Jarolim *et al.*, 1998; Yenchitsomanus *et al.*, 2003, 2005). Other *SLC4A1* mutations result in red blood cell abnormalities, HS and southeastern Asian ovalocytosis (Miraglia del Giudice *et al.*, 1997, Ranney *et al.*, 1990, Wrong *et al.*, 2002). Most mutant HS alleles generate unstable mRNA, and thus, reduced (or absent) mutant eAE1 polypeptide. Nevertheless, patients with HS have an apparently normal renal acidification phenotype (Alper, 2006). *SLC4A1* polymorphisms have also been described (Jarolim *et al.*, 1992, Miraglia del Giudice *et al.*, 1997), including the Diego blood group (Baleotti *et al.*, 2003), some of which are involved in the antigenicity of blood groups.

In this work, we present an analysis of the *SLC4A1* gene in three Mexican patients with HS, previously identified with combined AE1 deficiency. In Subject I, there was a 32% reduction in AE1 and 39% in the 4.2 protein, in Subject II, an 18.5% reduction in AE1, and in Subject III a 37% reduction in AE1 and 49% in spectrins.

The salting-out method (Miller *et al.*, 1988) was used for DNA extraction, and the polymerase chain reaction (PCR) for amplifying the promoter region and 20 exons (21 fragments) of the *SLC4A1* gene, this with the previously described primers and appropriate PCR conditions (Miraglia del Giudice *et al.*, 1997). Each PCR product included complete exon and splice sites, on which heteroduplex analysis was performed as previously described (Zhang and Minoda, 1996). Electrophoretically abnormal products were sequenced with an ABI-PRISM 310 sequencer, using a BigDye Terminator reaction kit (v. 3.1; Applied Biosystems, Foster City, CA, USA).

Heteroduplex analysis revealed six abnormal electrophoretic fragments in Subject I (exons 4, 11, 12, 13, 19, and 20), four in Subject II (exons 11, 13, 17, and 20) and six in Subject III (exons 3, 7, 9, 11, 14, and 16).

In subject I, two heterozygous substitutions were detected by DNA sequencing, namely a 166 A→G in exon 4 and a 2561 C→T in exon 19, corresponding to the known Memphis I (Lys56Glu) and Diego-a blood group (Pro854Leu) polymorphisms, respectively; these changes were identified in the subject's mother, also in heterozygosis. No further DNA changes were revealed on sequencing the remaining *SLC4A1* exons. In Subject II no changes were observed in all the 20 sequenced exons. Six exons with abnormal electrophoretic patterns were sequenced from Subject III, and a CCGG duplication of nucleotides 1885-1888 at exon 14 was found in the heterozygous state. The patient's father and brother did not carry this mutation, whereas the mother was not available for study purposes.

Two Memphis variants have been described. Memphis I (Lys56Glu) is relatively common in native Americans (frequency up to 25%), Japanese (29%) and African Americans (15%) (Ideguchi *et al.*, 1992, Jarolim *et al.*, 1992, Ranney *et al.*, 1990), besides Mexicans (11%) (Camacho-Torres *et al.*, 2006). The Memphis II variant carries Lys56Glu and Pro854Leu polymorphisms (Bruce *et al.*, 1994; Spring *et al.*, 1992). The Diego-a (Di^a^) allele (Pro854Leu) is the result of a 2561 C→T change in exon 19 (Bruce *et al.*, 1994), and is common in native South Americans (up to 54%), Mexicans (20.4%), Japanese (12%), Koreans (6.4%-14.5%) and Chinese (5%) but is rare in Caucasians (0.01%). It is considered a genetic marker for people of East Asian origin, therefore significant for anthropological studies (Baleotti *et al.*, 2003, Junqueira and Castilho, 2002). In most cases the Di^a^ allele (Pro854Leu) is linked to Memphis I polymorphism (Lys56Glu) (Bruce *et al.*, 1994; Spring *et al.*, 1992), although an unlinked form was observed in four out of 70 Amazonian Indians (Baleotti *et al.*, 2003). Studies in various populations are required to verify the occurrence of individuals with the 2561 C→T (Di^a^ allele) not linked to Memphis I polymorphism. The fact that both changes were maternally inherited in Subject I is suggestive that they are *in cis*, thus characterizing the Memphis II variant. Anyway, these polymorphisms are not related to the HS phenotype of Subject I. No HS causative *SLC4A1* mutation in this subject was observed, even after sequencing exons revealed as negative by heteroduplex analysis. Another gene might be contributing to the combined AE1 and 4.2 protein deficiency in this Subject (*e.g.*, *EPB42*). Nevertheless, a mutation, such as a large deletion and undetectable by sequencing, cannot be excluded, neither in this patient nor in Subject II, for whom no changes were revealed on sequencing all the *SLC4A1* exons. There are at least 11 *Alu* sequences which would facilitate mispairing and unequal crossing over.

In Subject III, the novel mutation 1885_1888dupCCGG apparently gives rise to the HS phenotype. The duplication in the sixth trans-membrane segment leads to a frameshift and predicts 57 different amino acids being encoded from codon 580 up to a stop codon at position 637. According to sequence analysis (NCBI Accession NM_000342.3), the truncated protein is probably unstable and without a normal function. A review of the literature (HGMD2009) showed 52 different *SLC4A1* mutations to be HS causative, and about 20 frameshift mutations, as that described herein.
